# Polyphenols extract from lotus seedpod (*Nelumbo nucifera* Gaertn.): Phenolic compositions, antioxidant, and antiproliferative activities

**DOI:** 10.1002/fsn3.1165

**Published:** 2019-08-09

**Authors:** Yingbin Shen, Yifu Guan, Xun Song, Jialiang He, Zhenxing Xie, Youwei Zhang, Hui Zhang, Dan Tang

**Affiliations:** ^1^ School of Public Health Dali University Dali China; ^2^ School of Life Sciences Guangzhou University Guangzhou China; ^3^ School of Chemistry and Chemical Engineering Guangxi University for Nationalities Nanning China; ^4^ School of Pharmaceutical Sciences, Health Science Center Shenzhen University Shenzhen China; ^5^ School of Food and Bioengineering Henan University of Science and Technology Luoyang China; ^6^ Basic School of Medicine Henan University Kaifeng China; ^7^ School of Food Science and Technology Jiangsu Food & Pharmaceutical Science College Huai'an China; ^8^ School of Food Science and Technology Jiangnan University Wuxi China; ^9^ Key Laboratory of Digital Quality Evaluation of Chinese Materia Medica of SATCM, Engineering Technology Research Center for Chinese Materia Medica Quality of Guangdong Province, School of Traditional Chinese Medicine Guangdong Pharmaceutical University Guangzhou China

**Keywords:** antioxidant, antiproliferation, lotus seedpod, phenolic profile, Yingbin Shen and Yifu Guan authors contribute equally to this paper

## Abstract

Seedpod, the nonedible portion of lotus (*Nelumbo nucifera* Gaertn.), was reported to be rich in polyphenols. The objective of this study was to investigate the major bioactive polyphenols of the lotus seedpods. The total polyphenol content (TPC) from ethanol extract of lotus seedpod (PELS) was found to be 34.23 μg gallic acid equivalents (GAE)/mg extract. Four polyphenolic compounds were identified in the PELS, comprised of one flavan‐3‐ol (catechin) and three flavonoids (kaemferol, quercetin and hyperoside). In vitro antioxidant and antiproliferative properties of the PELS were evaluated. PELS exhibited 89.38%, 99.82%, 68.25%, and 95.82% scavenging activities against 2,2‐diphenyl‐1‐picrylhydrazyl (DPPH), superoxide, hydroxyl, and 2,2ʹazinobis‐3‐ethylbenzothiazoline‐6‐sulfonic acid (ABTS) radicals, respectively, at 1.6 mg/ml. The Fe^3+^ reducing power of PELS was 0.605 at 0.32 mg/ml, which is comparable to glutathione (GSH). The PELS showed 31.79% metal chelating capacity and 87.79% inhibition of linoleic acid auto‐oxidation at 1.6 mg/ml. PELS showed cytotoxicity toward HepG2 and LNcap cell lines in vitro with IC_50_ values at 44.59 and 11.50 μg/ml, respectively. The findings of this study provide evidences that the inedible lotus seedpod could be a source for natural antioxidants and anticancer agents.

## 1 INTRODUCTION

Oxygen is vital for the sustenance of human and other aerobic organisms, and its depletion during cell metabolism produces by‐products such as reactive oxygen species (ROS). The main ROS produced by physical and chemical factors contain molecular oxygen (O_2_), superoxide anion radicals (O_2_^•−^), hydroxyl radicals (•OH), peroxyl radicals (RO_2_^•^), and hydrogen peroxide (H_2_O_2_). Excessive ROS could cause oxidative damage to DNA, proteins, and lipids resulting in some chronic diseases. Increasing evidences have shown that these ROS are involved in various serious pathological conditions including cancer, diabetes, liver injury, and age‐related neurodegenerative diseases (Liao et al., 2016; Schieber & Chandel, 2014).

Vitamins A, C, and E are the most commonly used diet supplement intake of antioxidants to prevent oxidative damage by ROS. Vegetables are full of vitamins and other antioxidants such as polyphenols which could reduce overproduction of ROS. Studies showed that dietary polyphenols as antioxidants can reduce the risk of chronic diseases related to ROS or oxidative stress, including diabetes mellitus (Wu et al., 2016), Alzheimer's disease (Tönnies & Trushina, 2017), and cancers (Sosa et al., 2013). Health concerns over human safety have increased interest in the development and utilization of natural, high potency, and low cost food‐derived antioxidants, to replace the current synthetic antioxidants.

*Nelumbo nucifera* Gaertn. is a valuable edible plant cultivated in China (Wu et al., 2004). All parts of this plant–roots, fruit, and seeds have various edible and medicinal uses in China (Mukherjee, Mukherjee, Maji, Rai, & Heinrich, 2009 et al.; Tian et al., 2018; Zhang, Cheng, Zhao, & Wang, 2017). In addition, the nonedible seedpod is reported to be rich in polyphenols and could be a potential source of new drug (Xiao et al., 2012). However, the antioxidant or antiproliferation activity of polyphenols from lotus seedpod has not been extensively studied. The objective of this study was to investigate the polyphenol composition and their antioxidative and antiproliferation activities.

## 2 MATERIALS AND METHODS

### 2.1 Materials and chemicals

Lotus seedpod was collected from Hongze Lake in Huai'an City, Jiangsu Province. The plant samples were dried at 40°C and ground into a fine, homogeneous powder using a combusted mortar and pestle. The DPPH, ferrozine, *N*‐methylphenazonium methyl sulfate (PMS), nitrotetrazolium blue chloride (NBT), butylated hydroxytoluene (BHT), nicotinamide adenine dinucleotide (NADH), and ABTS were commercially obtained from Sigma (Sigma).

### 2.2 Extraction of polyphenols from lotus seedpod

At room temperature, 5 g of lotus seedpod powder was soaked in 100 ml ethanol (50%) for 24 hr. The solid residue was filtered and further extracted once more under the same conditions. The resultant filtrates were combined and concentrated. Water was added to the concentrated residue and successively extracted with petroleum ether and ethyl acetate. The ethyl acetate fraction was used for LC‐MS analysis and bioassays.

### 2.3 Determination of TPC

The analysis of the TPC in PELS was performed based on the Folin–Ciocalteu method (Fogarasi, Kun, Tanko, Stefanovits‐Banyai, & Hegyesne‐Vecseri, 2015). Firstly, 0.2 ml of PELS (1 mg/ml in methanol) and 1.0 ml of Folin–Ciocalteu's phenol solution were mixed and incubated in a glass tube at room temperature for 10 min. 2.5 ml of 5% Na_2_CO_3_ was then added to the mixture, mixed, and added deionized water to make up to 10 ml. After 40 min, the absorbance was measured at 765 nm using a UV‐V double beam spectrometer (UV‐1700; Shimadzu). TPC was expressed as grams of GAE per 100 gram of dry weight (g GAE/100g DW). Gallic acid is standard phenolic compound, in which concentration ranges are 0–6.75 μg/ml.

### 2.4 Determination of total flavonoid content (TFC)

TFC in PELS was measured according to the previous assay with minor changes (Silva, Feliciano, Boas, & Bronze, 2014). In brief, 20 mg of PELS was dissolved in 10 ml of 50% aqueous methanol. Then, 300 μl of PELS solution, 3.4 ml of 30% methanol, 150 μl of 0.5 mol/L sodium nitrite, and 150 μl of 0.3 mol/L AlCl_3_⋅6H_2_O were added and mixed in a 10 ml test tube. After 10 min incubation, 1 ml of 1 M sodium hydroxide was added. The TFC was evaluated at 510 nm using a spectrophotometer and expressed as grams of rutin equivalents (RE) per 100 gram dry weight of extract. Rutin is reference compound, in which concentration ranges are 0–100 μg/ml.

### 2.5 Identification of polyphenols by HPLC‐DAD‐ESI‐MS

The methanol solution of PELS (100 μg/ml) was filtered by a 0.22‐μm syringe filter and analyzed by HPLC‐DAD‐ESI‐MS (Agilent) equipped with a C18 column (4.6 × 250 mm, 5 μm, Shimadzu). The mobile phase is acetonitrile (A) and water including 2% formic acid (v/v, B), which was delivered as follows: flow rate: 0.4 ml/min; 0 min, 15% (A); 1 min, 35% (A); 2 min, 40% (A); 5 min, 75% (A); 15 min, 15% (A); and 20 min, 15% (A). The spectral data were recorded using a DAD over a scanning wavelength ranging from 200 to 600 nm. An ESI ion source mass spectra were recorded in negative mode: fragmentor voltage, 100 V; nebulizing pressure, 25 psi; dry gas temperature, 300°C; capillary voltage, 2,500 V; and mass range, *m*/*z* 100–1000.

### 2.6 Evaluation of antioxidant activity

Antioxidant potential of PELS was, respectively, determined by DPPH radical, superoxide anion radical, hydroxyl radical, ABTS^·+^, metal chelating, and ferric reducing ability. The lipid peroxidation of PELS was also investigated. These results were compared with BHT, and the procedures are briefly explained as under.

#### 2.6.1 DPPH radical scavenging activity

Antioxidant potential of PELS was investigated using the DPPH radical scavenging assay published by Shimada, Fujikawa, Yahara, and Nakamura, (1992). 50 μl of undiluted sample solution was mixed with 0.2 ml of DPPH standard solution (24 mg/L, prepared by ethanol) in the 96‐well plates. After 30 min incubation, the absorbance was measured immediately at 517 nm using a spectrophotometer. The scavenging activity of PELS sample solution was calculated by applying the formula:

$$\text{DPPH}\;\text{radical}\;\text{scavenging}\;\text{activity}\left(\%\right)=\left[\frac{\text{Blank}\;\text{absorbance}-\text{Sample}\;\text{absorbance}}{\text{Blank}\;\text{absorbance}}\right]\times 100$$

#### 2.6.2 Scavenging activity on superoxide anion radical

The superoxide anion radical was generated using the PMS‐NADH‐NBT system previously described by Singh and Rajini (2004) with some modification. At room temperature, PELS (0.25–2.0 mg), NADH (73 μM), phosphate buffer (20 mM, pH 7.4), NBT (50 μM), and PMS (15 μM) were mixed and incubated for 5 min. The absorbances of the resultant reaction mixture were measured at 560 nm. The percentage inhibition of superoxide anion radical generation was calculated as follows:

$$\text{Inhibition}\left(\%\right)=\left[\frac{\text{Bank}\;\text{absorbance}-\text{Sample}\;\text{absorbance}}{\text{Bank}\;\text{absorbance}}\right]\times 100$$

#### 2.6.3 Scavenging activity on hydroxyl radical

The scavenging activity of PELS against the hydroxyl radical was investigated by an assay procedure published by Rodrigues et al. (Rodrigues et al., 2016), with some modification. Different concentrations of the test sample or reference compound were mixed with FeCl_3_ (100 μM), ethylenediaminetetraacetic acid (EDTA) (100 μM), 2‐deoxy‐2‐ribose (2.8 mM), KH_2_PO_4_‐KOH buffer (20 mM, pH 7.4), H_2_O_2_ (1.0 mM), and ascorbic acid (100 μM). The final volume of the mixtures was made up to 1 ml and then incubated for 1 hr at 37°C. 1 ml 2.8% trichloroacetic acid (TCA) and 1 ml 1% aqueous thiobarbituric acid (TBA) were added to 0.5 ml of the reaction mixture and incubated at 90°C for 15 min. The absorbance of the resultant reaction mixtures was measured at 532 nm after cooling. Results were expressed as percentage of inhibition, relative to a control sample.

#### 2.6.4 ABTS assay

The scavenging activity of the PELS against ABTS^·+^ was performed by Thana's method (Thana et al., 2008). ABTS (7 mM) was oxidized with potassium persulphate (2.45 mM) to obtain ABTS^·+^ radical. For this assay, 50 μL PELS was reacted with 200 μL of ABTS^·+^ radical. The reaction mixture's absorbance was measured at 734 nm after 10 min incubation.

$$\text{ABTS scavenging activity}\left(\%\right)=100\times \left(A_{\text{control}}-A_{\text{sample}}\right)/A_{\text{control}}$$

#### 2.6.5 Ferric reducing power assay

The reducing power of PELS was evaluated based on the method published by Borawska et al. (Borawska, Darewicz, Vegarud, & Minkiewicz, 2016). 0.5 ml phosphate buffer (pH 6.6, 0.2 mol/L) and 0.5 ml potassium ferricyanide (1%, w/v) were added to 0.2 ml of diluted PELS solution (1 mg/ml). After reacting for 60 min at 50°C, 0.5 ml trichloroacetic acid (10%, w/v) was added and centrifuged (800*g*, 5 min). 0.5 ml supernatant from the mixture was mixed with 0.5 ml distilled water and 0.1 ml ferric chloride (0.1%, w/v). The absorbance (700 nm) of the reaction mixtures was recorded after 15 min incubation. Reducing power was calculated as follows:

$$\text{Reducing power}=A\left(\text{sample}\right)-A\left(\text{control}\right)$$

#### 2.6.6 Metal ion chelating activity

The metal ion chelating activity of the PELS was analyzed by Giménez's method (Giménez, Alemán, Montero, & Gómezguillén, 2009). The change in sample's color was recorded on a spectrophotometer. The positive control was EDTA. Results were expressed as a percentage of the control, which is 100%.

#### 2.6.7 Linoleic acid assay

The inhibition of the auto‐oxidation and degree of oxidation of linoleic acid by PELS were evaluated using Chen's methods (Chen, Muramoto, & Yamauchi, 1995). Samples were dissolved in 2 ml of 0.1 mol/L phosphate buffer (pH 7.4) and then mixed with 2.0 ml of 2.5% linoleic acid in ethanol in test tubes, which were kept at 40°C in the dark. The total incubation time was 7 days. At regular intervals (24 hr), aliquots of the reaction mixtures were withdrawn with a microsyringe for measurement of the oxidation. The reference standard was BHT in the analysis. 8 ml of 75% ethanol, 100 μL of 30% ammonium thiocyanate, and 100 μL of 20 mmol/L ferrous chloride solution in 3.5% HCl were added to 100 μL of the reaction mixture. After 3 min reaction, absorbance of the colored solution at 500 nm was measured using a spectrophotometer. OD values (A_t_) were measured after every 24 hr. The antioxidant capacity of the sample was represented by the inhibition rate of oxidation at 144 hr, and the inhibition rate was calculated.

$$\text{Inhibition rate}\left(\%\right)=100\times {\left(A_{\text{control}}-A_{\text{sample}}\right)}_{t=144\;h}/A_{\text{control}}-A_{t=0\;h}$$

### 2.7 Antiproliferation properties

The HepG2 and LNcap cells were grown in Dulbecco's Modified Eagle's Medium (DMEM), which were supplemented with 10% fetal bovine serum, 50 units/mL streptomycin, and 100 units/mL penicillin in a humidified (5% CO_2_, 95% air) atmosphere at 37°C. The MTT cytotoxicity assay was performed according to the method (Zhang et al., 2016). Cells (1 × 10^4^ cells/well) were seeded for 12 hr in 96‐well plates. The cells were exposed to various concentrations of PELS for 24 hr and 48 hr. A 20 μl portion of MTT with 5 mg/ml in PBS was added into a well and incubated for another 4 hr. The solvent was removed, and dimethyl sulfoxide (DMSO) (150 μl/well) was added to dissolve the crystals with shaking for 10 min. The absorbance was measured at 570 nm by the microplate reader (BioTek). The inhibition rate was calculated using the following formula:

$$\text{Inhibition rate}\;\left(\%\right)=100\times \left(A_{\text{control}}-A_{\text{sample}}\right)/\left(A_{\text{control}}-A_{\text{blank}}\right)$$

The IC_50_ value was determined by nonlinear regression, type sigmoidal, analyzed using Graphpad Prism 5.0 software.

### 2.8 Statistical analysis

All the experiments were performed in triplicate, and the results are presented as the means ± *SD*. Duncan's multiple‐range test was used to analyze the significant differences, and a *p < *.05 was considered significant.

## 3 RESULTS AND DISCUSSION

### 3.1 Total phenols and flavonoids content

Natural polyphenols benefit human health (Ferrazzano et al., 2011). Reports indicate that lotus root was rich in various secondary metabolites such as polyphenols and flavonoids (Park et al., 2009). In this study, we determined the polyphenols and flavonoids contents of dry lotus seedpod extract (as depicted in Table 1). Our results show that TPC of PELS is higher in quantity as compared to phenolic content (22.5 mg GAE/g) in lotus cultivar from Korea (Park et al., 2009). The difference is probably caused by the different lotus species and extraction methods. The result of the TPC confirms that the extract is rich in phenolic contents.

**Table 1:** Contents of total phenolics and total flavonoids, and characterization of phenolic compounds in lotus seedpod by HPLC‐DAD‐ESI‐MS

Peak no.	Retention Time (min)	[M‐H]^‐^, *m/z*	Identity	Molecular Weight	Content g/100 g DW
1	2.26	593	Not determined	594	–
2	2.65	289	Catechin	290	8.27 ± 0.25
3	3.70	463	Hyperoside	464	5.51 ± 0.16
4	5.14	285	Kaempherol	286	1.24 ± 0.08
5	5.22	301	Quercetin	302	3.11 ± 0.11
TPC(Total phenol content, mg GAE/g DW)	–	–	–	–	34.23 ± 4.84
TFC (Total flavonoid content, g RE/100g DW)	–	–	–	–	25.12 ± 3.58

Note

Means not applicable.

### 3.2 LC/MS analysis of PELS

**Figure 1:**
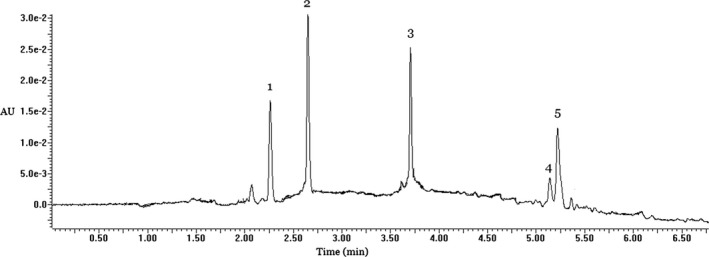
HPLC‐DAD‐ESI‐MS analysis of PELS

**Figure 2:**
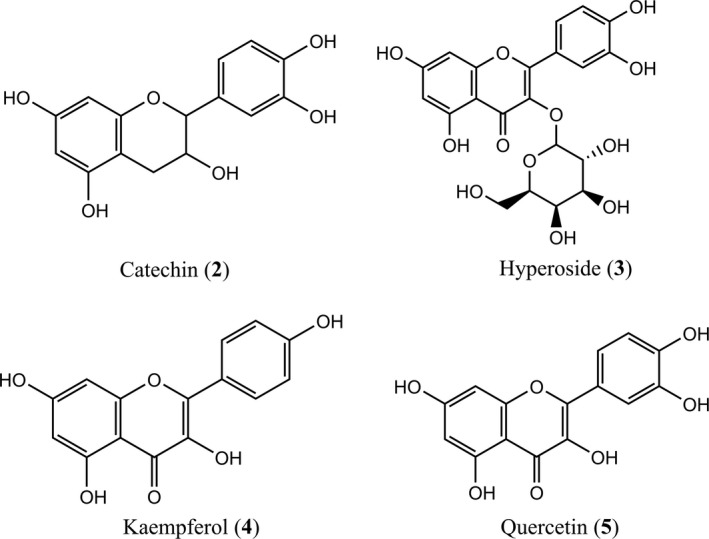
Chemical structures of compounds identified in the PELS

The ethyl acetate fraction was used to identify the polyphenols composition in PELS. As shown in Figure 1 and Table 1, five compounds were found according to the analysis of HPLC‐DAD/ESI‐MS. The *m*/*z* values of peaks 1, 2, 3, 4, and 5 in negative ESI‐MS mode ([M‐H]^‐^) were 593, 289, 463, 285, and 301. Peaks 2–5 were identified as catechin, hyperoside, kaempferol, and quercetin by comparing with the authentic standards, respectively (Figure 2). The identity of peak 1 remained inconclusive in the absence of a standard compound. The content of each identified compound was determined by HPLC‐DAD analysis (as shown in Table 1). Hyperoside was the main compound in PELS. To the best of our knowledge, reports on contents of single compound from lotus seedpot are limited. Our study allowed for the identification and content of polyphenols in the seedpod of *N. nucifera* Gaertn.

### 3.3 Antioxidant activity

#### 3.3.1 DPPH radical scavenging effect

**Figure 3:**
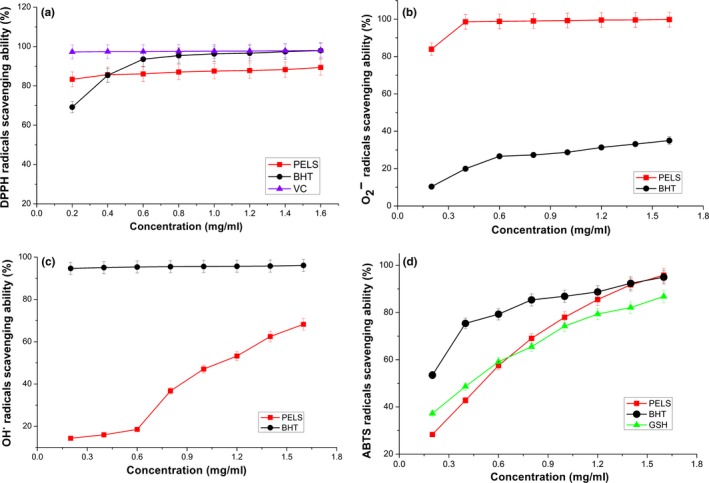
Scavenging effects of polyphenols extraction from lotus seedpod on DPPH radicals (a), superoxide anion radicals (b), hydroxyl radicals (c), and ABTS^•+^ radicals (d). Data represent the mean ± *SD* (*n* = 3)

DPPH is a simple method which is widely used to evaluate the antioxidant activities. Figure 3a shows the DPPH radical scavenging activity of ascorbic acid, BHT, and PELS. The effect of PELS on the DPPH radical increased from 83.36% to 89.38%, with increasing concentrations ranging from 0.2 to 1.6 mg/ml. Compared with the reference standards, the scavenging effect followed this order: ascorbic acid > BHT > PELS (98.08, 98.0, and 89.38, respectively) at 1.6 mg/ml.

The DPPH radical scavenging effect of PELS was reached above 80% at 0.2 mg/ml compared with control, together with increase in PELS concentration. We postulated that the polyphenols of PELS might contribute significantly to its scavenging activity according to the analysis of composition in the PELS. The active hydrogen atoms in the phenolic compound readily react with radicals and reduce the DPPH radical to DPPH‐H, thereby decreasing the absorbance of the solution. The hydrogen atom‐donating ability of compounds is often related to their antioxidant potential. The investigation reveals that PELS may be an excellent electron/hydrogen donator and could become an effective free radical scavenger.

#### 3.3.2 Superoxide anion radical scavenging effects

Superoxide anion radical is the single electron reduction product of oxygen molecule, which widely exists and is often continuously produced in normal physiologic reactions in the human body. Although it is not highly toxic to cells, its toxic effects cause adverse effects by producing hydrogen peroxide and highly reactive hydroxyl radicals (Xie & Chen, 2008). Polyphenols are recognized as great scavengers of superoxide anion radicals (Papuc, Goran, Predescu, Nicorescu, & Stefan, 2017). Figure 3b shows the percentage radical inhibition induced by PELS and positive control at 0.2–1.6 mg/ml. The PELS exhibited excellent scavenging activity (83.97%–99.82%) on the superoxide anion radicals at all the tested concentrations, and the scavenging effect was significantly (*p* < .05) higher than the reference (10.36%–35.01%) at the same concentration. The PELS extract showed strong superoxide anion radical scavenging activity (above 95% at 0.4 mg/ml).

It is probable that some active constituents, such as polyphenols, reacted readily with superoxide anion radicals and inhibited the formation of blue formazan in the reaction mixture, resulting into a decrease in the absorbance. The results indicated that the PELS possessed significant superoxide anion radical scavenging activity and could be a good source of natural antioxidants.

#### 3.3.3 Hydroxyl radical scavenging activity

Hydrogen peroxide can cross cell membranes and react with Fe^2+^ and Cu^2+^ ions to obtain hydroxyl radicals. This may be the origin of many of its toxic effects (Qi et al., 2005). In this study, the hydroxyl radicals were used to evaluate the hydroxyl radical scavenging activity of PELS. As depicted in Figure 3c, the PELS exhibited moderate hydroxyl radical scavenging activity (14.39%–68.25%) in a concentration‐dependent manner. Comparable scavenging effect on hydroxyl radical to that of BHT would encourage the use of PELS as a safe natural‐based health food supplement at higher concentrations.

#### 3.3.4 ABTS^·+^ radical scavenging effects

As depicted in Figure 3d, the PELS showed ABTS^·+^ radical scavenging activity of 28.33%–95.82%, in a concentration‐dependent manner (0.2–1.6 mg/ml, *p* < .05). Although the scavenging effect of PELS was less efficient than that of BHT and GSH at lower concentrations, it exhibited a higher activity than that of GSH at 0.8 mg/ml. The ABTS^·+^ scavenging effect of PELS was the most potent of all the tested compounds at 1.6 mg/ml. The order of activity was as follows: PELS > BHT > GSH (95.82, 94.96, and 86.81%, respectively).

Stable ABTS radicals were generated by donating the labile hydrogen atom of antioxidants to peroxyl radical; therefore, antioxidants can terminate the radical reaction. We postulated that some polyphenols in the PELS, which may be electron or hydrogen donors, played the role of “radical terminator” by quenching and eliminating the ABTS^·+^, thereby decreasing the absorbance of the reaction solution. Moreover, active groups that donate electrons in the polyphenols structure may contribute to the radical scavenging activity. All these results indicate that the PELS may be an excellent electron donator with comparable effects to those of the synthetic compounds, BHT and GSH, and likely possesses strong ABTS^·+^scavenging ability in vivo.

#### 3.3.5 Reducing power assay

**Figure 4:**
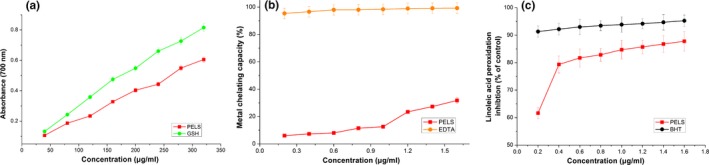
The reducing power (a), metal chelating capacity (b), inhibition of linoleic acid peroxidation (c) at various concentrations of polyphenols from lotus seedpod

The relationship between antioxidant activity and reducing power has been described (Duh, Tu, & Yen, 1999). The higher reducing power of antioxidants often has strong ability to donate electron. Therefore, reducing power is normally used to forecast the antioxidant capacity. The reducing power of PELS (Figure 4a) with GSH as a reference standard revealed that it had a significant (*p* < .05) concentration‐dependent effect, that was 0.605 at 320 μg/ml. Furthermore, the reducing power of PELS was very close to that of GSH at the same concentration. The results elucidate that the PELS are good electron and hydrogen donors and can convert free radicals to more stable products. Thus, the PELS could be used as a potent antioxidants.

#### 3.3.6 Metal chelating capacity

Transition metals have been reported to catalyze the formation of the first free radicals required to initiate the propagation of the radical chain reaction in lipid peroxidation (Mathew & Abraham, 2006). Therefore, we examined chelating capacity of PELS against Fe^2+^, because it is the most effective pro‐oxidant found in food (Lin, Wei, & Chou, 2006). As shown in Figure 4b, the formation of the ferrozine‐Fe^2+^ was not completed in the presence of PELS. This suggests that PELS competed with ferrozine for chelating the ferrous iron, and this effect increased in a concentration‐dependent manner. Existence of metal ions, especially Fe^2+^, will accelerate lipid peroxidation exponentially in food systems. Therefore, the metal chelating capacity that PELS possessed can indirectly retard lipid peroxidation although the metal chelating capacity of PELS was lower than that of EDTA.

#### 3.3.7 Antioxidant activity in linoleic acid system

Lipid peroxidation is often thought to occur *via* radical‐mediated abstraction of H atoms from methylene carbons in polyunsaturated fatty acids (Rajapakse, Mendis, Byun, & Kim, 2005). It is probable that some phenolic compounds in the PELS, which are excellent hydrogen donators, more readily supply hydrogen atoms than the linoleic acid. This protects against peroxidation. Therefore, the antioxidant effects of PELS against the peroxidation of linoleic acid were evaluated (Figure 4c, reference standard: BHT). PELS significantly inhibited the peroxidation of linoleic acid at the tested range. At concentrations of 0.8, 1.0, 1.2, and 1.6 mg/ml, PELS showed 82.86%, 84.73%, 85.72%, and 87.79% inhibition rate, respectively. But, the inhibitory effect of the PELS was slightly lower than that of BHT.

As we know, essential fatty acids including linoleic acid and arachidonic acid possess important physiological functions and are precursors of some physiological active substances, but they are very susceptible to ROS and free radicals because of their structure of carbon–carbon double bond. Therefore, oxidation loss of essential fatty acids in food may lead to synthesis deficit of physiological active substances and cause physiological malfunction or even some related diseases. According to this study, it is known that the PELS can inhibit peroxidation of linoleic acid effectively. Hence, it is deduced that the PELS may protect essential fatty acids in food from oxidation induced by ROS and free radicals. All these results indicate that the PELS possessed considerable inhibitory effects against auto‐oxidation of linoleic acid and, therefore, could have considerable potential application in the oil and fat processing industry.

### 3.4 Antiproliferative activity of the PELS

**Figure 5:**
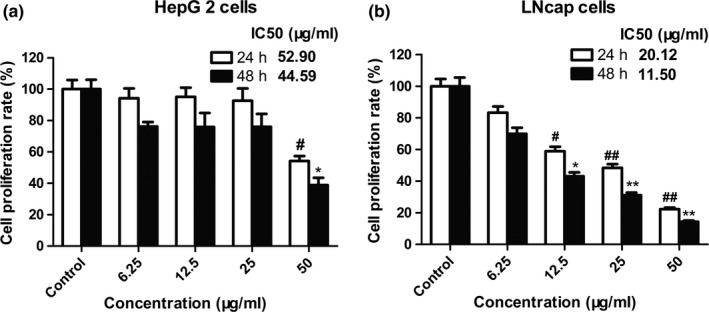
Cell growth inhibition in HepG2 and LNcap cells PELS‐exposed. Cell proliferation is expressed as a percentage of the maximum value compared with the control cells (DMSO‐treated). Dose‐dependent decrease in values was higher in LNcap than in HepG2 cells. Each column refers to the mean ± *SEM*. *n* = 3. *p < .05 and ** p < .01 compared with corresponding DMSO‐treated control cells after 24 hr. *^#^p* < .05 and *^##^ p* < .01 compared with corresponding DMSO‐treated control exposed to 48 hr

MTT assay was conducted on HepG2 and LNcap cells to evaluate the antiproliferative effect of PELS. All tested cells were treated at various concentrations of PELS (6.25, 12.5, 25, and 50 μg/ml) for 24 and 48 hr, and the results were illustrated in Figure 5. As revealed by the growth curves, PELS only showed slight antiprolierative activity against HepG2 cells with 54.21% inhibition at 50 μg/ml compared with control after 24 hr. After a period of incubation of 24 and 48 hr, PELS showed a dose‐dependent inhibition from 12.5 to 50 μg/ml on LNcap cells (Figure 5b). Cell treatment with 25.0 μg/ml PELS for 24 hr reduced cell growth by 50% and treatment for 48 hr reduced by 70%. The maximum effect was obtained with 50 μg/ml PELS, which demonstrates PELS cytotoxic effect to LNcap cells.

Zhao group reports the antiproliferative activity of alkaloids from lotus seedpot (Zhao et al., 2016), but none targeted toward polyphenols against HepG2 and LNcap cells. The antiproliferative effect of PELS may be due to the polyphenol constituents, based on the results of our component analysis. Our study suggests that polyphenols from lotus seedpot further extended the healthcare benefits of *N. nucifera* Gaertn.

## 4 CONCLUSIONS

The present study provides new information about the antioxidative and antiproliferative properties of the lotus seedpod extract and can be considered of practical interest. In this study, catechin, hyperoside, kaempferol, and quercetin were identified as the major constituents from PELS. The PELS showed significant antioxidant properties and potent antiproliferative activity, which may be correlated with polyphenol constituents. Our study provides evidences that the lotus seedpod can be used as a potent bioactive source of natural antioxidant and anticancer agents.

## CONFLICT OF INTEREST

The authors declare that they do not have any conflict of interest for this work.

## ETHICAL REVIEW

This study does not involve any human or animal testing.

## INFORMED CONSENT

Written informed consent was obtained from all study participants.

## References

[fsn31165-bib-0001] Borawska, J. , Darewicz, M. , Vegarud, G. E. , & Minkiewicz, P. (2016). Antioxidant properties of carp (*Cyprinus carpio* L.) protein ex vivo and in vitro hydrolysates. Food Chemistry, 194, 770–779. 10.1016/j.foodchem.2015.08.075 26471617

[fsn31165-bib-0002] Chen, H. M. , Muramoto, K. , & Yamauchi, F. (1995). Structural analysis of antioxidative peptides from Soybean.beta.‐Conglycinin. Journal of Agriculture and Food Chemistry, 43, 574–578. 10.1021/jf00051a004

[fsn31165-bib-0003] Duh, P. D. , Tu, Y. Y. , & Yen, G. C. (1999). Antioxidant activity of water extract of Harng Jyur (*Chrysanthemum morifolium* Ramat). LWT‐Food Sci. Tech., 32, 269–277. 10.1006/fstl.1999.0548

[fsn31165-bib-0004] Ferrazzano, G. F. , Amato, I. , Ingenito, A. , Zarrelli, A. , Pinto, G. , & Pollio, A. (2011). Plant polyphenols and their anti‐cariogenic properties: A review. Molecules, 16, 1486–1507. 10.3390/molecules16021486 21317840 PMC6259836

[fsn31165-bib-0005] Fogarasi, A. L. , Kun, S. , Tanko, G. , Stefanovits‐Banyai, E. , & Hegyesne‐Vecseri, B. (2015). A comparative assessment of antioxidant properties, total phenolic content of einkorn, wheat, barley and their malts. Food Chemistry, 167, 1–6. 10.1016/j.foodchem.2014.06.084 25148951

[fsn31165-bib-0006] Giménez, B. , Alemán, A. , Montero, P. , & Gómezguillén, M. C. (2009). Antioxidant and functional properties of gelatin hydrolysates obtained from skin of sole and squid. Food Chemistry, 114, 976–983. 10.1016/j.foodchem.2008.10.050

[fsn31165-bib-0008] Liao, W. , Chen, L. , Ma, X. , Jiao, R. , Li, X. , & Wang, Y. (2016). Protective effects of kaempferol against reactive oxygen species‐induced hemolysis and its antiproliferative activity on human cancer cells. European Journal of Medicinal Chemistry, 114, 24–32. 10.1016/j.ejmech.2016.02.045 26974372

[fsn31165-bib-0009] Lin, C. H. , Wei, Y. T. , & Chou, C. C. (2006). Enhanced antioxidative activity of soybean koji prepared with various filamentous fungi. Food Microbiology, 23, 628–633. 10.1016/j.fm.2005.12.004 16943061

[fsn31165-bib-0010] Mathew, S. , & Abraham, T. E. (2006). In vitro antioxidant activity and scavenging effects of Cinnamomum verum leaf extract assayed by different methodologies. Food and Chemical Toxicology, 44, 198–206. 10.1016/j.fct.2005.06.013 16087283

[fsn31165-bib-0011] Mukherjee, P. K. , Mukherjee, D. , Maji, A. K. , Rai, S. , & Heinrich, M. (2009). The sacred lotus (*Nelumbo nucifera*) ‐ phytochemical and therapeutic profile. Journal of Pharmacy and Pharmacology, 61, 407–422. 19298686 10.1211/jpp/61.04.0001

[fsn31165-bib-0012] Papuc, C. , Goran, G. V. , Predescu, C. N. , Nicorescu, V. , & Stefan, G. (2017). Plant Polyphenols as Antioxidant and Antibacterial Agents for Shelf‐Life Extension of Meat and Meat Products: Classification, Structures, Sources, and Action Mechanisms. Comprehensive Reviews in Food Science and Food Safety, 16, 1243–1268. 10.1111/1541-4337.12298 33371586

[fsn31165-bib-0013] Park, Y. S. , Towantakavanit, K. , Kowalska, T. , Jung, S. T. , Ham, K. S. , Heo, B. G. , … Gorinstein, S. (2009). Bioactive compounds and antioxidant and antiproliferative activities of Korean white lotus cultivars. Journal of Medicinal Food, 12, 1057–1064. 10.1089/jmf.2009.0018 19857070

[fsn31165-bib-0014] Qi, H. , Zhang, Q. , Zhao, T. , Chen, R. , Zhang, H. , Niu, X. , & Li, Z. (2005). Antioxidant activity of different sulfate content derivatives of polysaccharide extracted from *Ulva pertusa* (Chlorophyta) in vitro. International Journal of Biological Macromolecules, 37, 195–199. 10.1016/j.ijbiomac.2005.10.008 16310843

[fsn31165-bib-0015] Rajapakse, N. , Mendis, E. , Byun, H. G. , & Kim, S. K. (2005). Purification and in vitro antioxidative effects of giant squid muscle peptides on free radical‐mediated oxidative systems. Journal of Nutritional Biochemistry, 16, 562–569. 10.1016/j.jnutbio.2005.02.005 16115545

[fsn31165-bib-0016] Rodrigues, M. J. , Neves, V. , Martins, A. , Rauter, A. P. , Neng, N. R. , Nogueira, J. M. F. , … Custodio, L. (2016). In vitro antioxidant and anti‐inflammatory properties of *Limonium algarvense* flowers' infusions and decoctions: A comparison with green tea (*Camellia sinensis*). Food Chemistry, 200, 322–329. 10.1016/j.foodchem.2016.01.048 26830595

[fsn31165-bib-0017] Schieber, M. , & Chandel, N. S. (2014). ROS function in redox signaling and oxidative stress. Current Biology, 24, R453–462. 10.1016/j.cub.2014.03.034 24845678 PMC4055301

[fsn31165-bib-0018] Shimada, K. , Fujikawa, K. , Yahara, K. , & Nakamura, T. (1992). Antioxidative properties of xanthan on the autoxidation of soybean oil in cyclodextrin emulsion. Journal of Agriculture and Food Chemistry, 40, 945–948. 10.1021/jf00018a005

[fsn31165-bib-0019] Silva, S. D. , Feliciano, R. P. , Boas, L. V. , & Bronze, M. R. (2014). Application of FTIR‐ATR to Moscatel dessert wines for prediction of total phenolic and flavonoid contents and antioxidant capacity. Food Chemistry, 150, 489–493. 10.1016/j.foodchem.2013.11.028 24360480

[fsn31165-bib-0020] Singh, N. , & Rajini, P. S. (2004). Free radical scavenging activity of an aqueous extract of potato peel. Food Chemistry, 85, 611–616. 10.1016/j.foodchem.2003.07.003

[fsn31165-bib-0021] Sosa, V. , Moline, T. , Somoza, R. , Paciucci, R. , Kondoh, H. , & Leonart, M. E. (2013). Oxidative stress and cancer: An overview. Ageing Research Reviews, 12, 376–390. 10.1016/j.arr.2012.10.004 23123177

[fsn31165-bib-0022] Thana, P. , Machmudah, S. , Goto, M. , Sasaki, M. , Pavasant, P. , & Shotipruk, A. (2008). Response surface methodology to supercritical carbon dioxide extraction of astaxanthin from *Haematococcus pluvialis* . Bioresource Technol., 99, 3110 10.1016/j.biortech.2007.05.062 17643298

[fsn31165-bib-0023] Tian, W. , Zhi, H. , Yang, C. , Wang, L. , Long, J. , Xiao, L. , … Zheng, J. (2018). Chemical composition of alkaloids of Plumula nelumbinis and their antioxidant activity from different habitats in China. Industrial Crops and Products, 125, 537–548. 10.1016/j.indcrop.2018.09.045 PMC6240636 30480072

[fsn31165-bib-0024] Tönnies, E. , & Trushina, E. (2017). Oxidative stress, synaptic dysfunction, and alzheimer's disease. Journal of Alzheimer's Disease, 57, 1105–1121. 10.3233/JAD-161088 PMC5409043 28059794

[fsn31165-bib-0025] Wu, S. , Sun, C. , Cao, X. , Zhou, H. , Hong, Z. , & Pan, Y. (2004). Preparative counter‐current chromatography isolation of liensinine and its analogues from embryo of the seed of *Nelumbo nucifera* GAERTN. using upright coil planet centrifuge with four multilayer coils connected in series. Journal of Chromatography A, 1041, 153–162. 10.1016/j.chroma.2004.05.003 15281264

[fsn31165-bib-0026] Wu, Y. Q. , Reece, A. , Zhong, J. X. , Dong, D. Y. , Shen, W. B. , Harman, C. R. , & Yang, P. X. (2016). Type 2 diabetes mellitus induces congenital heart defects in murine embryos by increasing oxidative stress, endoplasmic reticulum stress, and apoptosis. American Journal of Obstetrics and Gynecology, 215(3), 366.e1–366.e10. 10.1016/j.ajog.2016.03.036. 27038779 PMC5260663

[fsn31165-bib-0027] Xiao, J. S. , Xie, B. J. , Cao, Y. P. , Wu, H. , Sun, Z. D. , & Xiao, D. (2012). Characterization of Oligomeric Procyanidins and Identification of Quercetin Glucuronide from Lotus (*Nelumbo nucifera* Gaertn.) Seedpod. Journal of Agriculture and Food Chemistry, 60, 2825–2829. 10.1021/jf205331e 22369273

[fsn31165-bib-0028] Xie, Y. S. , & Chen, X. M. (2008). Epidemiology, major outcomes, risk factors, prevention and management of chronic kidney disease in China. American Journal of Nephrology, 28, 1–7. 10.1159/000108755 17890852

[fsn31165-bib-0029] Zhang, L. , Cheng, Z. , Zhao, Q. , & Wang, M. (2017). Green and efficient PEG‐based ultrasound‐assisted extraction of polysaccharides from superfine ground lotus plumule to investigate their antioxidant activities. Industrial Crops and Products, 109, 320–326. 10.1016/j.indcrop.2017.08.018

[fsn31165-bib-0030] Zhang, Y. , Zhou, X. , Tao, W. , Li, L. , Wei, C. , Duan, J. , … Ye, X. (2016). Antioxidant and antiproliferative activities of proanthocyanidins from Chinese bayberry (Myrica rubra Sieb. et Zucc.) leaves. Journal of Functional Foods, 27, 645–654. 10.1016/j.jff.2016.10.004

[fsn31165-bib-0031] Zhao, X. , Feng, X. , Peng, D. G. , Liu, W. W. , Sun, P. , Li, G. J. , … Song, J. L. (2016). Anticancer activities of alkaloids extracted from the Ba lotus seed in human nasopharyngeal carcinoma CNE‐1 cells. Experimental and Therapeutic Medicine, 12, 3113–3120. 10.3892/etm.2016.3727 27882126 PMC5103758

